# Neurosurgery Residency Program Directors’ Perspectives: A Scoping Review of Program Director Survey Responses

**DOI:** 10.7759/cureus.90910

**Published:** 2025-08-24

**Authors:** Andie Conching, Sabrina Chriqui, Hayley Granberg, Saarang Patel, Mohammad F Khan, Lauren Stone, Nolan Brown, Martin Pham

**Affiliations:** 1 Neurosurgery, University of Hawaii, John A. Burns School of Medicine, Honolulu, USA; 2 Neurosurgery, University of California, San Diego School of Medicine, San Diego, USA; 3 Neurosurgery, Seton Hall University, South Orange, USA; 4 Neurological Surgery, Indiana University School of Medicine, Indianapolis, USA; 5 Neurological Surgery, University of California, Irvine, Irvine, USA

**Keywords:** medical students, neurosurgery, program director, residency, survey

## Abstract

Entrance to neurological surgery residency is highly competitive due to the large number of applicants vying for a limited number of spots. The process has become even more competitive in recent years, with a significant increase in applicants but a consistent number of available residency positions. Program director (PD) surveys offer valuable insights into the selection process and expectations for neurosurgical residency, guiding prospective candidates to navigate the challenging training path. We conducted a three-database scoping review confined to the last 20 years to compile all available PD survey results, excluding those released through the official National Resident Matching Program (NRMP) data. Studies were screened and selected according to specific inclusion and exclusion criteria. The selected articles were evaluated for survey year, distribution method, response rate, question type, number of items, and content. Most importantly, survey responses were detailed and summarized. Nineteen PD survey studies were eligible for inclusion in the present review. These revealed substantial diversity in survey structure and topics addressed over the past 20 years, with response rates averaging 54%. Studies focused on PD perspectives on applicant evaluation criteria, residency training factors, and the impact of COVID-19 on the application process. Key findings included the importance of the interview process, United States Medical Licensing Exam (USMLE) Step 2 scores, and letters of recommendation in resident selection, concerns about the impact of the USMLE Step 1 transitioning to pass/fail, and the need for enhanced research opportunities and mentorship to increase interest in neurosurgery. Residency training studies highlighted challenges related to duty hour restrictions, the importance of surgical simulation for resident education, and variations in subspecialty training experiences. The impact of COVID-19 on residency applications posed challenges for certain applicant groups during virtual interviews, and barriers remain in the post-pandemic era. Recent studies provide an in-depth look at PD perspectives on essential factors in the neurosurgery residency match, the impact of COVID-19 on recruitment, and the current state of training. Selection criteria still heavily rely on USMLE scoring, letters of recommendation, and interviews, with debates arising from the shift to pass/fail Step 1 scoring. Challenges in evaluating candidates, concerns about training quality, and the effects of the 80-hour workweek mandate are ongoing issues, while research participation is encouraged to enhance academic productivity. The pandemic has influenced the application process and led to mixed outcomes and financial barriers for some applicants despite the return to in-person interviews. These findings illustrate the continuous evolution of neurosurgical residency programs in response to various challenges and reforms.

## Introduction and background

Gaining entrance to a neurological surgery residency involves a highly competitive sub-specialty match [1]. One of the primary drivers of the high selectivity intrinsic to the neurosurgery match is high applicant demand, which substantially outweighs the supply of residency positions [2]. It is no secret that entrance into neurosurgery residency is merely the beginning of a long, arduous training that is challenging intellectually, emotionally, and even physically [3,4]. In recent years, the process has become even more competitive. For example, during the 2023 cycle, the total number of applicants to neurosurgery residency increased by 31.9% from the 2020 cycle [2]. As the growing number of candidates continues to exceed the limited training positions in neurosurgery, prospective applicants need to understand the resident selection process and training expectations that follow.

One approach to gaining more insight into the neurosurgical residency selection process is through program director (PD) surveys, which typically gather data on different aspects of a residency program in a given specialty, including curriculum, resources, and program outcomes. By sampling responses from the individuals who develop the training curriculum and support resident culture, these surveys have proven invaluable as references that neurosurgical candidates can turn to for guidance.

The objective of the present study is to summarize via scoping review the existing body of neurosurgery PD surveys to consolidate data into a single resource that is readily accessible to prospective candidates interested in pursuing neurosurgery.

## Review

Methods

Literature Search

This study compiled all available PD surveys (excluding those released through the official National Resident Matching Program (NRMP) match data) and was performed in accordance with the Preferred Reporting Items for Systematic Reviews and Meta-Analyses (PRISMA) guidelines for scoping reviews “extension” (PRISMA-ScR), with the exception that the study’s protocol is not available for access via online registry [5]. A three-database query was conducted using PubMed, Google Scholar, and Scopus to identify all studies publishing survey study data obtained from neurosurgery residency PDs. The search was performed on January 8, 2024, using the following Boolean search terms over the preceding 20 years: (neurosurg*) AND (program director OR PD) AND (survey).

Study Selection Process

All studies returned by the search were screened according to title and abstract, following removal of duplicates. Remaining studies then entered the full-text review phase, which was performed independently by three authors (A.C., S.C., L.S.). When disagreements regarding inclusion arose, the senior author (M.P.) served as arbiter in facilitating a final decision regarding inclusion.

Study Selection Criteria

To qualify for inclusion in the present review, studies must have been (1) available in full-text as (2) peer-reviewed publications reporting (3) primary data obtained through PD surveys. Articles were reviewed for relevance and excluded if (1) they were non-primary survey studies, (2) were not available in English, (3) did not report data relevant to neurosurgery residency candidate selection or residency training, or (4) were limited to NRMP-only survey data.

Data Compilation

The selected articles were evaluated for survey year(s), distribution method, response rate, survey instrument type(s), number of survey items, and survey instrument content. Most importantly, survey responses were detailed and summarized.

Results

Search Results

**Figure 1:**
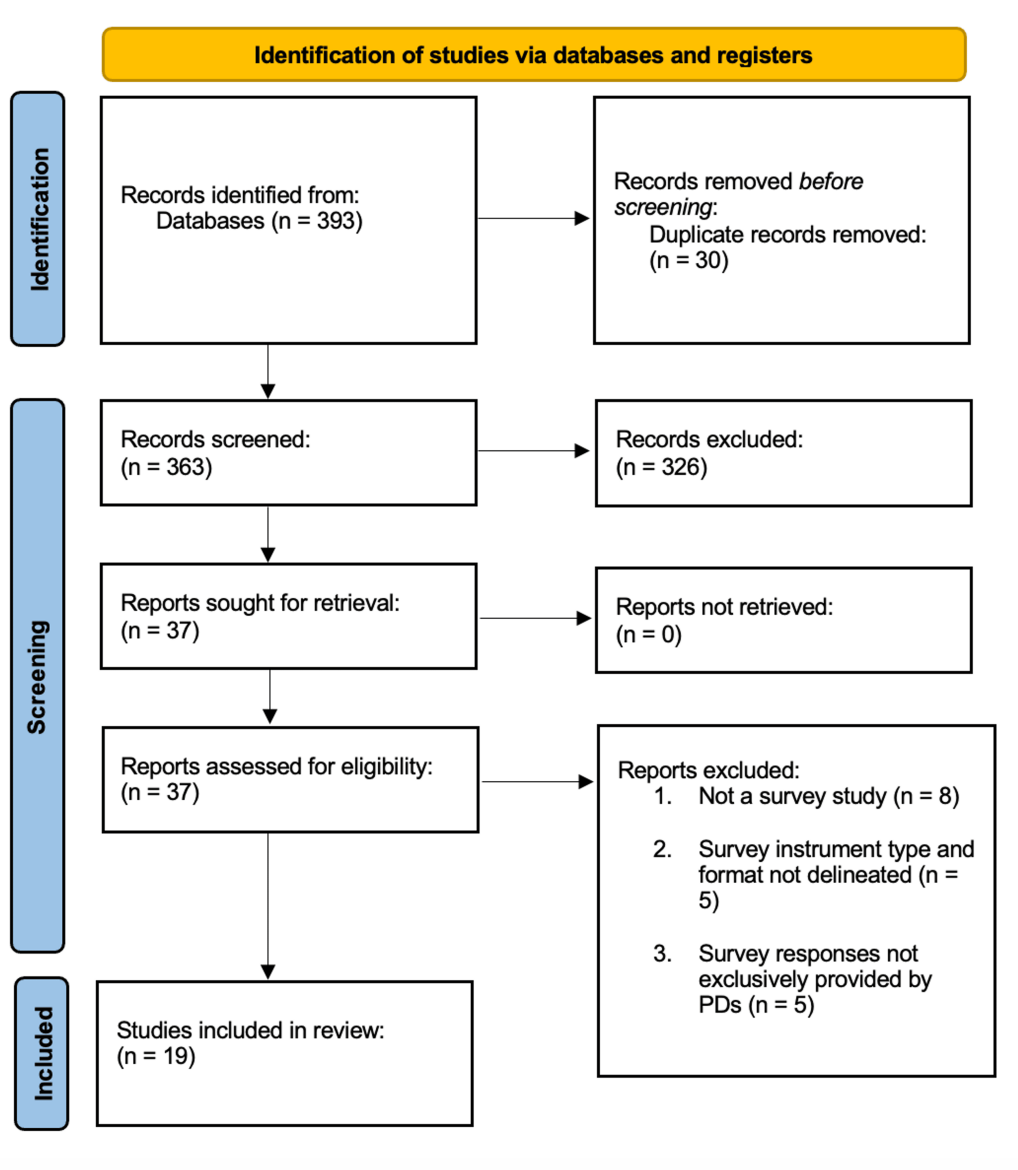
PRISMA diagram detailing the study selection process. PRISMA: Preferred Reporting Items for Systematic Reviews and Meta-Analyses.

Our three database queries (n = 363 unique entries) and systematic screen (n = 344 studies excluded) returned 19 studies deemed eligible for inclusion in the present review (Figure 1). Among these 19 studies, three primary topical themes were identified: residency candidate selection (n = 6), effects of the COVID-19 pandemic on the residency selection process (n = 2), and resident training factors (n = 11).

Overall, there was substantial diversity in the structure, format, and range of subtopics addressed among the 19 survey studies (Table 1). Surveys (results detailed in Supplementary Table) ranged from six to 49 questions, and the average response rate was 54.0% (range: 27.0% to 92.7%). Survey instruments most frequently consisted of (1) non-ordinal, multiple-choice questions or (2) Likert scales. Other instruments used less frequently included (3) rank-order responses and (4) open-ended, free-response questions. Collectively, the surveys were conducted over the preceding 20 years (2004-2023).

**Table 1:** Program director survey details and summary. ACGME: Accreditation Council for Graduate Medical Education; USMLE: United States Medical Licensing Examination; PD: program director; APD: associate program director; PC: program coordinator; AMA: American Medical Association; AANS: American Association of Neurological Surgeons; DC: department chair; LOR: letter of recommendation; CK: clinical knowledge; EBM: evidence-based medicine.

Year	Author	Title	Survey year and method	Response rate	Question type	Number of survey items	Survey content
COVID-19 pandemic							
2021	Romano et al. [3]	Optimizing the residency application process: insights from neurological surgery during the pandemic virtual application cycle	2021, electronic distribution via academic email addresses, 2 weeks after the 2021 neurosurgery residency match results, with 2 additional reminders and a second distribution 1 month later	Program administrators (PAs): 67.0% (77/115 PAs). Program leaders (PLs): 51.7% (119/230 PLs)	Multiple choice, free response	49 items (PAs), 63 items (PLs)	Demographic/program information. Opinions on optimal sub-internship experiences. Interview administration. Perceived positive and negative effects of the virtual interview format. Ability to assess applicant personality. Views of standardized application policies. Methods for improving the match cycle
2022	Jimenez et al. [4]	Perceptions of the virtual neurosurgery application cycle during the coronavirus disease 2019 (COVID-19) pandemic: a program director survey	Emailed with follow-up sent 3 weeks later	27% (38/141 PDs and APDs) 32 PDs + 6 APDs	Multiple choice, rank order, free response	30 items	Demographic/program information. Factors used to review applicants in the interview cycle. Perceptions of applicants and applicant engagement. Perception of standardized letters and interview questions. Effect of virtual interview format on various stakeholders. Efficacy and future outlook for virtual residency interview format
Admissions							
2020	Field et al. [6]	Selection of neurological surgery applicants and the value of standardized letters of evaluation: a survey of United States program directors	November 2018, emailed to the program director of each U.S. neurosurgical residency program and kept open for 6 months with 3 reminders sent (1 to program coordinator)	47% (53/113 programs), 89% PDs, 7% DCs, 4% APDs	Rank order, Likert scale (5-point), multiple choice, open-ended	16 items	Demographic/program information. Rank the elements of the current residency application. Support or nonsupport of potential objective elements in a standard letter of evaluation (SLOE). Assessment of current LOR and potential implementation of SLOE. Textbox for further comments
2014	Al Khalili et al. [7]	Programs selection criteria for neurological surgery applicants in the United States: a national survey for neurological surgery program directors	August 2011, mailed to ACGME-accredited neurosurgery programs with follow-up by phone call and email	46% (46/100 programs)	Free response, multiple choice, Likert scale (4-point)	23 items	Demographic/program information: program description, hospital- vs. university-based, number of applicants, number of accepted applicants, intention to increase program size (resident numbers), number of foreign medical graduates accepted. Interview process: number of candidates invited, length of interview process, who conducts interviews, and factors evaluated in the interview. Decision process: who finalizes the rank order list, the importance of each factor in resident selection, minimum cut-off scores. Retrospective view of past decisions: degree of satisfaction of PD with performance of residents and applicant pool
2020	Huq et al. [8]	Perceived impact of USMLE Step 1 pass/fail scoring change on neurosurgery: program director survey	Emailed to each PD and APD with a follow-up email 1 week later	52% (59/119 PDs and 16/26 APDs)	Multiple choice, rank order, free response, Likert scale	23 items	Demographic/program information. USMLE Step 1 information. Global perceptions about change in USMLE Step 1 scoring. Perceived predictive ability of USMLE Step 1 on neurosurgery resident performance. Perceptions of who would benefit or suffer from the scoring change. Impact on and advice for future applicants
2020	Kumar et al. [9]	Characterizing the effect of pass/fail U.S. Medical Licensing Examination Step 1 scoring in neurosurgery: program directors' perspectives	Emailed to ACGME-accredited neurosurgery training programs, PDs were invited as part of a recently reported national survey distributed to 30 specialties; open for a 4-week period	48.5% (48/99 PDs)	Likert scale (3-point)	19 items	Perspectives regarding the change of USMLE Step 1 scoring to pass/fail. Perspectives/changes that will be made because of the USMLE Step 1 new scoring format.
2020	Lubelski et al. [10]	Improving medical student recruitment to neurosurgery	January 2018	27% (30/110 programs)		Not mentioned	Demographic/program information. Exposure to neurosurgery in formal education. Mandatory clinical rotations. Neurosurgery outreach. Research opportunities. Sub-internships and electives
2022	Stein et al. [11]	Assessing the impact of changes to USMLE Step 1 grading on evaluation of neurosurgery residency applicants in the United States: a program director survey	Emailed to PDs, PCs, and ADPs compiled from AMA Fellowship and Residency Electronic Interactive Database and cross-referenced with AANS residency directory, with follow-up email at 2 and 6 weeks	30% (35/117 programs)	Free response, multiple choice, rank order	15 items	Demographic/program information. Opinions on USMLE Step 1, Step 2 CK, and possible changes in reporting after scoring change. Ranking a list of 16-17 factors considered when assessing applicants for candidacy by order of importance before and after the USMLE Step 1 scoring change
Resident training							
2004	Lunsford et al. [12]	Survey of United States neurosurgical residency program directors	March 2003, mailed with follow-up email reminder	79.1% (72/91 PDs)	Multiple choice	31 items	Manpower issues (neurosurgeons, residents), training process, residency review committee (RRC) governance issues
2005	Cohen-Gadol et al. [13]	Resident duty hours reform: results of a national survey of the program directors and residents in neurosurgery training programs	July-September 2003, emailed to PDs (could not find 2 PD addresses), with reminder requests in August and September	45% (42/93 PDs)	Multiple choice, open-ended	23 items	Impact of ACGME guidelines on continuity of patient care and resident education. Number of residents in the program. Employment of physician extenders, trauma coverage, and the number of cases handled by residents before and after the implementation of ACGME guideline changes
2014	Daniels et al. [14]	The current state of United States spine surgery training: a survey of residency and spine fellowship program directors	December 2012, an online questionnaire was emailed to PDs with a reminder email follow-up 1 week after distribution	45.1% (46/102 PDs)	Likert scale (5-point), multiple choice	42 items	Opinions on contemporary spine surgery training
2013	Ganju et al. [15]	The role of simulation in neurosurgical education: a survey of 99 United States neurosurgery program directors	Emailed directly to PDs with a reminder 1 week later	53.5% (53/99 PDs)	Likert scale (5-point), multiple choice	14 items	Clinical impact of simulation. Role of simulation in academia. Willingness to invest time and money in simulator practice. Device best suited for simulation
2023	Kilbourn et al. [16]	Incorporating simulation into the neurosurgical residency curriculum: a program director survey	Feb-June 2022, REDCap, sent link to survey, email reminders distributed as needed	42% (48/113 PDs)	Branching logic, multiple choice, check all that apply	15 items	Demographic/program information. Assess how surgical simulation is currently being used by neurosurgical residency programs. Simulation characteristics. Role of simulation. Impact of COVID-19
2014	Kshettry et al. [17]	The role of laboratory dissection training in neurosurgical residency: results of a national survey	Electronic cover letter and survey sent to PDs, with three attempts made with approximately 2-week intervals between attempts	65% (65/100 PDs)	Multiple choice	11 items	Prevalence of laboratory dissection training. Logistics of laboratory dissection. Resident participation, curriculum, and educational impact
2015	Haglund et al. [18]	Difficult conversations: a national course for neurosurgery residents in physician-patient communication	Electronically disseminated to PDs by the existing LISTSERV	85% (87/102 PDs)	Multiple choice, check all that apply	6 items	Current practices and gaps in communication training in graduate medical education (GME) programs. Feedback on current teaching and evaluation practices addressing trainee communication competence. Usefulness of new education tools (videos and specific workshop design) in individual training programs and in helping to meet new educational milestones
2018	Gupta et al. [19]	Neurosurgical resident error: a survey of U.S. neurosurgery residency training program directors’ perceptions	January 2017, distributed by email, kept open for 3 weeks with 2 reminder emails sent at the end of week 1 and week 2	28.7% (31/108 programs)	Free response, multiple choice, check all that apply	19 items	Demographic/program information. Perceived incidence of various types of errors (procedural/surgical errors, diagnostic errors, medical errors). Clinical outcomes after resident error (little to no consequence, transient injury, permanent injury, death). Percent of cases believed to involve surgical error that required additional/repeat surgery. Steps taken by the residency to address errors for first or repeat offenders. Relative effect of postgraduate year and ACGME duty-hour restrictions on error rate. Perceived efficacy of the ACGME American Board of Neurological Surgery’s Milestone project in reducing the rate of resident error
2019	Limoges et al. [20]	Pediatric neurosurgery training during residency in the United States: a program director survey	Distributed by email	77% (86/111 PDs)	Free response, multiple choice, check all that apply	25 items	Structural nature of pediatric training at an individual neurosurgery program. Demographic/program information. Resident demographics. Pediatric-specific education structure, rotations, and case volume
2019	Lepard et al. [21]	Neurosurgical resident research education: a survey of United States residency program directors	April 10, 2017 - January 24, 2018, emailed to PDs, then the chairperson was contacted if PD was not responsive; continued techniques until an over 90% response rate was achieved	92.7% (102/110 programs; 4/102 completed by chairperson)	Multiple choice, check all that apply	20 items	Extent of focused EBM education in neurosurgical training programs in the U.S.
2020	Miller et al. [22]	International electives in neurological surgery training: a survey of program directors from Accreditation Council for Graduate Medical Education-approved neurological surgery programs	Distributed with REDCap to program coordinators (PCs) for PDs to complete, PCs contacted another time, with surveys distributed 2 more times directly to PDs who had not responded	44% (50/113 programs)	Branching logic, Likert scale (5-point), open-ended	10-15 items (branching)	Demographic/program information. Availability and logistics of training opportunities in developing countries. Interest in starting international electives. Perceived barriers and values of international rotation. Whether the program encourages resident participation in international rotations

Program Selection Criteria and Applicant Evaluation

Six studies focused on the criteria used to select neurosurgical residents [6-11]. For example, Al Khalili and colleagues underscored the importance of the interview process, United States Medical Licensing Exam (USMLE) Step 1 scores, and letters of recommendation (LORs) in resident selection [7]. Ultimately, Al Khalili’s study found that PDs view applicant standardization as integral to the residency selection process and desire a replacement for the traditionally scored USMLE Step 1 in its absence. In echoing Al Khalili’s findings, two additional studies reported PD perspectives on the conversion of Step 1 to pass/fail [8,9]. PDs indicated near-unanimous opposition to the change from a scored to a pass/fail Step 1. In surveying for alternative means by which PDs might standardize applicants, Stein and colleagues reported that PDs might consider factors such as medical school ranking and USMLE Step 2 scores more critically during the applicant evaluation process [11]. Collectively, Huq et al. and Kumar et al. joined Stein et al. in reporting PD consensus that USMLE Step 2 scores, LORs, and publication quantity will inevitably gain increased significance in the candidate selection process [8,9,11]. Furthermore, Field and colleagues highlighted PD preferences for enhancing the objectivity of the application process through standardized LORs due to the change of USMLE Step 1 to pass/fail [6,7].

Finally, when looking into medical student recruitment, Lubelski et al. reported that early exposure to neurosurgery, access to research opportunities, mentorship, and involvement in neurosurgery interest groups increase the number of medical students pursuing a career in neurosurgery and, thus, the volume of neurosurgery residency applicants [10].

Neurosurgical Residency Training and Curriculum

Eleven studies investigated various aspects of neurosurgical training and curriculum; many of them date back to the mid-2000s and early 2010s when debate regarding resident work hour restrictions became a hot topic [12-22]. For example, Lunsford and colleagues examined the impact of the 80-hour workweek mandate on resident learning [12]. Their survey was indicative of significant PD concern regarding its negative effects on training quality. Similarly, Cohen-Gadol et al.’s survey study echoed the PD perspective that duty hour reform negatively impacts resident training by interfering with the continuity of patient care (due to increased frequency of patient hand-offs) [13]. In the aftermath of residency duty hour restrictions, Daniels and colleagues noted that while most residents still averaged over 450 spine cases, PDs and fellowship directors felt that fellowship training was a soft requirement for trainees pursuing careers in spinal deformity surgery [14]. Ultimately, findings from this survey study suggested the need for additional post-residency training to achieve competency.

Additionally, in two separate studies, both Ganju et al. and Kilbourn et al. addressed the importance of alternative means for residents to gain operative proficiency while under duty hour reductions [15,16]. Their findings pointed to the potential importance of surgical simulation. This importance was signified by the fact that many programs reported the incorporation of new surgical simulation modalities into resident training despite financial and spatial constraints.

Another important residency training feature assessed via survey was variation in resident subspecialty training experiences. Although one might expect neurosurgical training to be relatively uniform across programs, Limoges and colleagues found significant inter-institutional variation in pediatric neurosurgery training, as reported by PDs [20]. Lastly, two studies reported PD survey results related to resident research education and elective opportunities. Lepard et al. noted that most programs facilitate resident research through structured curricula, journal clubs, and dedicated research time, while Miller et al. discovered that few programs have established global neurosurgery electives [21,22]. Though they found this to be the case, the PDs Miller et al. surveyed indicated a strong interest in organizing global neurosurgery rotations involving international resident experiences despite the presence of approval and funding challenges [22].

Impact of COVID-19 on Residency Applications

Two studies covered the influence of the COVID-19 pandemic on the residency application process, providing insights into applicant groups facing heightened challenges in the post-pandemic era [3,4]. Both studies noted a surge in applicant volumes during the virtual application cycles and recognized potential disadvantages of the virtual interview format for doctors of osteopathic medicine (DOs) and international medical graduates (IMGs). For example, Romano et al. highlighted that students from prestigious programs and those pursuing a doctor of medicine (MD) degree gained advantages from the transition to virtual interviews [3]. By contrast, Jimenez et al. noted that PD self-reported applicant evaluation criteria remained largely unchanged compared to pre-pandemic cycles [4].

Discussion

Resident Selection and the Neurosurgery Match

Resident selection criteria for neurosurgery residency programs have shifted in response to changes such as the conversion of USMLE Step 1 to pass/fail scoring and the COVID-19 pandemic. PDs express uncertainty about whether the current application process sufficiently evaluates applicant quality. Furthermore, there is notable dissatisfaction among PDs with virtual interviews and concern that new metrics, such as Step 2 scores, LORs, and research publication quantity, will be disproportionately emphasized.

Medical Student Recruitment

One study investigated the state of pre-residency medical student recruitment to neurosurgery. In a survey of 30 PDs, Lubelski et al. found that high-matching programs (programs that attain high neurosurgery match rates and volumes among their medical students) are more likely to have a neurosurgery interest group (100% versus 65%, p = 0.02) as well as greater integration of formal research requirements into their medical student curriculum (77% versus 24%, p = 0.01) when compared to low-matching programs [10]. Additionally, a greater percentage of high-matching programs had formal neurosurgery mentorship programs in place (69% versus 30%, p = 0.06) [10]. Early exposure, research opportunities, and mentorship programs are crucial for attracting neurosurgery applicants, and high-matching programs are more likely to integrate formal requirements featuring these factors.

Importance of PD-Reported Factors in Residency Candidate Selection

Al Khalili et al.’s 23-question survey, distributed in August 2011, assessed PD perspectives on the interview process, the rank list decision process, and PD satisfaction with past candidate selection decisions [7]. The survey had a 46% response rate and queried PDs through free-response, multiple-choice, and Likert scale questions [7]. Overall, results indicated that PDs judged the interview process, USMLE Step 1 scores, and LORs as the most important factors in applicant assessment when forming the rank list. Naturally, these findings became increasingly pertinent when USMLE Step 1 transitioned to pass/fail. When candidate selection criteria were rated on a four-point scale indicating importance (4 = most important), the interview process was rated as the most important overall factor (3.80 ± 0.65). Following interview performance, the USMLE Step 1 score had the second-highest average rating (3.58 ± 0.54), and LORs were the third-highest (3.56 ± 0.54). PDs reported that interviews are most often conducted by PDs (100% respondents), with varying involvement of residents and other faculty members, and range from four to eight hours in length (52.17%) [7]. These findings highlight the critical role of interviews and standardized test scores in maintaining an objective residency selection process, especially in light of changes to the USMLE Step 1 scoring system.

When asked to rank factors as positive, neutral, or negative, PDs recognized honesty (100%), interactions with residents at dinner (95%), energy (90%), decision-making (67.5%), and confidence (65%) as positive interviewee qualities, while aggression and anxiety were both viewed negatively (70% and 85%, respectively) [7]. Regarding their program’s resident selection process, 60.87% of PDs indicated that they were “very satisfied,” while 36.95% selected “somewhat satisfied,” and only 2.17% indicated that they were “not satisfied.” Though this is the case, it still stands that the majority of PDs admitted that they would not choose all the previously selected residents from the past five years.

Six years following Al Khalili et al.’s publication of their PD survey results, Field and colleagues released the results of their 16-item PD survey [6]. Their survey assessed elements of the resident selection process with an added focus on the perceived utility of the LOR and on the prospect of implementing a standardized letter of evaluation (SLOE) [6]. This survey had a 47% response rate and included a mixture of multiple-choice, rank-order, Likert scale, and open-ended questions. Respondents were asked to rank seven residency application components from 1 being the most important to 7 being the least important. The three application components with the highest rankings (average rank ± SD) were the LOR (2.96 ± 1.52), USMLE Step 1 score (2.86 ± 1.44), and candidate interview performance (2.0 ± 1.40). These were the same three components identified as most important in Al Khalili et al.’s survey [6,7].

While emphasizing their perception of the importance of LORs, Field and colleagues reported PD consensus that familiarity with the LOR author strengthens the value of the recommendation (100%) and that LORs offer a realistic way to compare applicants (81%) [6]. There was also agreement that there would be value in increasing the objective nature (78%) and interrater reliability (84%) of the LOR, thereby increasing its standardization.

Interestingly, the statement least agreed with was that LORs are valuable predictors of future resident performance (51%). On the open-ended portion of the survey, some respondents indicated uncertainty as to what extent other letter writers are honest in their evaluation of their own students [6]. The varied perspectives on the LOR and the support for SLOE highlight the need for more standardized, objective, and reliable evaluation tools in the residency selection process to ensure fair and comprehensive candidate assessments.

When asked about implementing an SLOE, 65% of respondents were in support of doing so [6]. Interestingly, this was also the statement that received the most responses of “strongly disagree,” indicative of the polarizing nature of SLOEs. When PDs were provided with a list of proposed questions about an applicant that could be featured on SLOEs, the questions most favored for inclusion concerned communication skills (88%), initiative (88%), work ethic (88%), and professionalism (86%). PDs also responded positively (85%) to the inclusion of a component of the SLOE that would ask writers to rank the tier of applicants based on several qualities. In their analysis, they noted that respondents who strongly agreed with increasing LOR objectivity and ranking applications based on the applicant’s perceived communication skills were more likely to be in support of incorporating the SLOE (p < 0.001; r^2^ = 0.580). However, this support did not translate to the overall rating of the LOR’s value as “high” (p = 0.962). In their open responses about the SLOE, respondents acknowledged that this new component might risk “introducing a new variable that would take some time to understand,” thereby introducing a new “learning curve.” The mixed reactions to the SLOE proposal and the emphasis on specific qualities in candidate evaluations suggest the need for thoughtful implementation of standardized tools that can accommodate diverse perspectives while enhancing the objectivity of the selection process.

Step 1 Pass/Fail

Three studies discussed the implications of the change in Step 1 scoring to a pass/fail format that was announced in 2020 and took place in 2022 [8,9,11]. Both Huq et al. and Kumar et al. reported that a majority of PDs expressed disagreement (79% and 78.7%, respectively) with the binary Step 1 scoring change [8,9]. The disagreement highlights the concern among PDs about losing a critical standardized metric that was heavily relied upon for comparing applicants objectively.

Huq and colleagues found that 77% of responding PDs “always” screened applicants by Step 1 scores [8]. Respondents also indicated strong disagreement with the statement that the Step 1 scoring change would have a positive impact on neurosurgery (72%) or the field of medicine (67%) [8]. Regarding the impact on trainees, 64% of respondents felt that the USMLE score was a strong predictor of resident success within their program. Perspectives on the impact of the change on PD’s ability to project candidate performance on the American Board of Neurological Surgeons (ABNS) written exam scores were mixed. Additionally, 73% of PDs predicted that the Step 1 scoring change would increase the number of neurosurgery applicants.

Both Huq et al. and Kumar et al. reported that PDs believed that the Step 1 scoring change would impact different types of applicants in contrasting ways [8,9]. Specifically, Huq et al. found that PDs believed students from prestigious medical schools would benefit (57%), and students from less prestigious schools would be disadvantaged (60%) [9]. Kumar et al. reported the PD sentiment that medical school reputation would become more important in resident selection (71%) and that IMGs would face more challenges than United States medical graduates (63.0%) as a result of the scoring change [8,9].

Finally, both studies addressed the fact that the majority of residency programs now expect to use USMLE Step 2 clinical knowledge (CK) to fill the role that USMLE Step 1 served in standardizing applications [8,9]. Huq et al. found that 49% of respondents believed Step 2 would “become the new Step 1,” while Kumar et al. found that 87.5% of PDs would increase emphasis on Step 2 CK scores [8,9]. These results led to the overall recommendation that applicants take Step 2 CK prior to the Electronic Residency Application Service (ERAS) submission because programs would eventually make (and now have made) this a requirement. The transition to emphasizing USMLE Step 2 reflects the need for a new standardized metric but also raises concerns about whether it can effectively replace Step 1 in evaluating applicants.

Stein et al.’s 2022 survey of 35 PDs found that most PDs anticipated USMLE Step 2 scores, class rank, and away rotations becoming the new standards used to determine applicant desirability after the conversion of Step 1 to pass/fail [11]. In regards to the American Board of Neurological Surgeons (ABNS) written board examination, 80.0% of respondents felt that Step 1 scores were accurate predictors of the future ability of a resident to pass the exam, while only 45.7% felt that Step 2 scores would be predictive of neurosurgery written board performance [11]. This is again reflective of the PD perspective that Step 1 scores represented a highly useful metric for stratifying applicants. Overall, the mixed confidence in Step 2 CK scores as a substitute for Step 1 highlights ongoing uncertainties and the need for additional reliable metrics to ensure a fair and comprehensive evaluation process.

As a result, a major focus of Stein et al.’s survey was identifying the factors that would be most heavily weighted when assessing applicant competitiveness [11]. Respondents were given a list of 17 factors and asked to rank them in order of importance. Before the Step 1 grading change, Step 1 score, LORs, and number of research items (abstracts, presentations, and publications) were identified as the most important factors, in that order. After the Step 1 scoring change, the order of importance of the top three factors shifted to LORs, research items, and USMLE Step 2 CK scores [11]. When considering factors both within and outside the top three, USMLE Step 2 CK scores, class rank, and away rotation performance significantly increased in importance in the absence of Step 1 scores [11]. Additionally, the importance of extracurricular involvement and leadership activities, as well as Alpha Omega Alpha membership, was de-emphasized following the scoring change [11].

COVID-19 Pandemic

In 2020, the COVID-19 pandemic led to the transition of away rotations (excepting the single externship allotted to each applicant) and interviews to a virtual format in light of travel restrictions [23-26]. Two surveys assessed PDs' feelings on the changes made due to pandemic-era restrictions.

Mignucci-Jimenez et al. reported a significant increase in the number of ERAS applications between the 2019-2020 and 2020-2021 cycles [25]. Romano et al. reported that the virtual interview was perceived by PDs to cause less financial and time burdens for candidates than conventional cycles [3]. On the other hand, PDs indicated that the virtual interview limited the ability of selection committees to assess the fit and personality of incoming candidates. Interestingly, the virtual interview format may have resulted in significant increases in the average number of applicants interviewed by each program (45.2 in 2020-2021 versus 39.9 in 2019-2020; p = 0.0003) as well as the number of interview sessions held (3.8 in 2020-2021 versus 3.3 in 2019-2020; p = 0.0003). Romano’s survey also indicated that the virtual match process was perceived as beneficial to students from more prestigious medical schools. On the other hand, the virtual cycle was viewed as a detriment to students from less prestigious medical schools (including DO schools), as external rotations for these individuals are presumably even more important than they are for candidates in prestigious and/or well-connected programs [3]. Last, Romano et al.’s survey addressed sub-internship logistics. When asked about the optimal length of home and external sub-internships, 93.3% of PDs favored one four-week home sub-internship with one four-week away rotation, and 68.9% of respondents favored two four-week external rotations.

Training

Eleven of the surveys address neurosurgical residency training over the period ranging from 2004 to 2023. Survey areas of interest included PD perception of neurosurgery manpower, program relationships with the Residency Review Committee (RRC), changes in training after the resident duty hour reform, resident error, and the value and use of different modes of learning in neurosurgical education.

Manpower and Work Hours

In 2004, Lunsford et al.'s survey suggested that 54.9% of PDs felt that there were too few residents at their institution, 50% of whom would increase the number of trainees by one every other year if permitted [12]. A total of 52.8% of respondents felt that the number of practicing neurosurgeons in the United States was too low, with 38.6% reporting unfilled neurosurgical staff positions in their regions. Only 4.4% felt that the RRC had an excellent grasp on present and future neurosurgical demands, and 67.6% felt that the RRC should have the authority to set the number of neurosurgical residents in training in the US. Moreover, 42.3% of respondents felt that the impact of the Accreditation Council for Graduate Medical Education (ACGME) 80-hour work week for neurosurgical trainees seriously detracted from resident education, whereas 9.9% felt that it enhanced overall resident education. Cohen-Gadol et al.’s study specifically focused on duty hour restrictions, revealing that most programs reported employment of ancillary healthcare professionals after the duty hour requirement went into effect [13]. Overall, 79% of PD respondents in Cohen-Gadol et al.’s survey felt that the ACGME guidelines negatively impacted their training program, especially with respect to the continuity of patient care. PDs also felt that their chief residents were exposed to fewer complex cases. These findings highlight significant concerns about the adequacy of neurosurgical manpower and the adverse effects of duty hour restrictions on training quality, suggesting the need for reevaluation of current policies to better balance education and workload.

Skills Augmentation

Three studies addressed neurosurgical skills augmentation via simulation and laboratory dissection. PDs from Ganju et al.’s survey believed that simulation would improve patient outcomes and viewed it as an effective supplement to conventional training [15]. While few thought that simulators replace aspects of neurosurgical training, 60% of PDs reported that they moderately agreed that resident use of simulators to practice neurosurgical techniques would improve skills in the operating room, and 58% reported that if made available, they would encourage residents to practice skills in a simulator, while another 38% reported they would mandate it. In terms of financial backing, 30% of respondents stated that they would invest more than $10,000 on simulators, while another 32% reported that they would spend between $5,000 and $10,000 toward this end. According to a recent study, the emphasis of PDs on surgical simulation would be well-received by neurosurgical residents [26].

Although PDs ranked cadaver practice as the least preferred medium for skill augmentation in Ganju et al.’s study, Kshettry et al. found that 93.8% currently incorporate laboratory dissection into their neurosurgery residency curriculum [17]. Approximately half of the PDs surveyed reported integrating physical models and/or computer-based virtual reality simulation into their curriculum, with the most commonly reported simulations involving endoscopy (50.8%), microvascular anastomosis (50.8%), spinal approaches (88.5%), spinal instrumentation (80.3%), and cranial approaches (100%). Additionally, 89.2% of PDs favor the idea of a widespread initiative geared toward developing a universal curriculum for neurosurgical residency laboratory dissections. Moreover, 95.4% of PDs feel that laboratory dissection is an integral component of neurosurgical residency training. There is a growing recognition of the importance of simulation and dissection in enhancing microsurgical skills, leading to advocacy for increased investment and integration of these methods into neurosurgical training programs.

Resident Error

Gupta and colleagues assessed PD perceptions on the frequency, type, causes, and clinical outcomes of errors made in training [19]. The median annual incidence of resident errors was three per year, ranging from one to 24 per year. The most frequently reported error type (48.4%, range 10% to 100%) was a surgical error resulting in no permanent injury. The most commonly reported cause of error was inexperience (45.9%, 0.0%-100.0%), followed by resident mistakes despite adequate training (35.5%, 0.0-100.0%). In regard to the latter, 74.2% of PDs attributed these errors to residents from lower postgraduate levels. Only 6.5% of PDs noted a correlation between a reduction in duty hour limitations and the incidence of resident error. The prevalence and causes of resident errors highlight the critical need for improved training methods and supervision to mitigate inexperience-related errors and enhance patient quality.

Training Curriculum

Five of the studies addressed elements of academic and clinical training, including aspects of competencies, curriculum, pursuit of fellowships, and options for international electives. Lepard and colleagues assessed evidence-based medical education curricula in neurosurgical residency programs [21]. This survey had the highest response rate of all included surveys (92.7%), with 96.1% of programs indicating that they have a regularly scheduled journal club. Additionally, 58.4% of programs reported having a formal curriculum in place for residents to learn key research methods and evidence-based medicine/quality improvement measures, with 57.3% reported having an annual research publication requirement built into the residency curriculum. Finally, 88.1% of respondents reported having protected research time ranging anywhere from seven to 24 months.

In addition to protected research time, programs have also begun integrating protected years for residents to pursue enfolded fellowships in specific subspecialties. Considering the increasing prevalence of enfolded fellowships, the role of the post-residency fellowship has naturally become a topic of debate. Daniels et al. evaluated opinions on contemporary spine surgery training and the need for post-residency fellowships [14]. In their survey, Daniels et al. reported that a substantial portion of PDs surveyed (36.6%) reported residents performing over 450 spine cases during residency. In turning attention from spine surgery to pediatrics, Limoges and colleagues evaluated resident exposure to pediatric neurosurgery [20]. Their survey results revealed wide variation across program curricula and that most programs (90.7%) had four or fewer pediatric neurosurgery faculty. Nonetheless, the vast majority (76.7%) of programs reportedly include specific pediatric neurosurgery rotations at their institutions. These typically occur during the postgraduate year 3, and nearly half (48.2%) last between four and six months.

With the popularity of global neurosurgery on the rise, Miller and colleagues surveyed PDs to learn more regarding international electives during neurosurgery residency [22]. Most programs reported not having an established international elective (74%), citing funding (51.4%), the RRC approval process (32.4%), resident call conflicts (27%), and the challenge of establishing an international partnership (27%) as key limitations to these electives. Half of the residencies without electives (54%) reported interest in starting an international elective. Perceived benefits of this learning opportunity included increased cultural awareness, exposure to unique pathologies, ingenuity, and greater physical examination and diagnostic skills.

Limitations

We acknowledge several limitations to the present review. First, while this study specifically sought to incorporate PD survey responses, the responses provided by just a single representative of a program (even an individual as integral as the PD) may or may not be reflective of a program’s overall stance. Additionally, the average response rate across administered neurosurgical PD surveys was 52.3%. With inconsistent participation, response bias is more likely to have had an effect on results. Low response rates introduce a higher probability that more extreme viewpoints are captured, and these views may not be representative of the perspective shared by the majority of the population surveyed. Furthermore, heterogeneity in the methods used across studies can serve as a source of potential bias. Of note, surveys differed in their method of distribution and follow-up (mail vs. email), which may have resulted in varying levels of engagement. Additionally, it is important to note that Likert scales can introduce the bias of central tendency, in which respondents may avoid extreme answer choices, or the social desirability bias, in which respondents may list answers they believe are socially acceptable rather than reflective of their true opinions. Each of these biases affects the validity and reliability of survey instruments, necessitating caution in drawing definitive conclusions from survey results reported in our study.

## Conclusions

Taken together, the collective findings reported in the present study provide a comprehensive overview of up-to-date PD perspectives regarding factors most (and least) important in the residency match, the impact of the COVID-19 pandemic on the residency recruitment process, and the current state of neurosurgery residency training and curriculum. In terms of the NRMP match, PDs indicated that selection criteria remain heavily reliant on USMLE scoring (i.e., using Step 2 as a cutoff), LORs, and interviews. Additionally, the COVID-19 pandemic has reshaped the residency match process, resulting in mixed effects that favor certain applicant demographics while potentially placing others at a disadvantage. With the return to in-person interviews, financial barriers are again resurfacing as significant challenges for socioeconomically disadvantaged applicants to overcome. Altogether, the findings reported in the present study highlight neurosurgical residency PDs’ ongoing adaptations to educational reforms, pandemics, and other external challenges that have required inventiveness and creativity on their part to overcome. Ultimately, we hope that this study will serve as an accessible, comprehensive resource that can contribute to a greater understanding of the evolving landscape of neurosurgical training.

## Appendices

**Table 2:** Program director survey results and findings (supplemental). PDs: program directors; USMLE: United States Medical Licensing Examination; DO: doctor of osteopathic medicine; IMG: international medical graduate; ACGME: Accreditation Council for Graduate Medical Education; EBM: evidence-based medicine; ABNS: American Board of Neurological Surgery; LOR: letter of recommendation; ERAS: Electronic Residency Application Service.

Year	Author	Title	Findings
2014	Al Khalili et al. [7]	Programs selection criteria for neurological surgery applicants in the United States: a national survey for neurological surgery program directors	Interview, USMLE Step 1, and LORs ranked highest. Fewer than 40 interviews and emphasis on extramural activities linked to higher PD satisfaction. Aggression and anxiety are viewed negatively. Honesty, decision-making, and confidence are viewed positively
2020	Field et al. [6]	Selection of neurological surgery applicants and the value of standardized letters of evaluation: a survey of United States program directors	The interview, USMLE Step 1 score, and LOR were ranked as the most important aspects of the resident application. The personal statement was ranked as the least important aspect. Most PDs supported more objective LORs. Opinions split on standardization due to concerns about loss of personalized content
2020	Lubelski et al. [10]	Improving medical student recruitment to neurosurgery	Higher match rates are tied to early exposure, neurosurgery interest groups, and formal research requirements. Exposure varied widely across institutions
2020	Huq et al. [8]	Perceived impact of USMLE Step 1 pass/fail scoring change on neurosurgery: program director survey	Most PDs opposed change, believing it harms applicant evaluation, disadvantages less prestigious schools, and IMGs. Many expect Step 2 to take on greater importance; most advise taking it before ERAS
2020	Kumar et al. [9]	Characterizing the effect of pass/fail U.S. Medical Licensing Examination Step 1 scoring in neurosurgery: program directors' perspectives	Most PDs disagree with binary Step 1 scoring, citing that it will make the comparison of students and the application process more difficult. The majority of PDs indicate that more emphasis will be placed on Step 2 and school reputation. Few PDs think that changing Step 1 to pass/fail will improve equity or well-being; most expect it to disadvantage IMGs.
2022	Stein et al. [11]	Assessing the impact of changes to USMLE Step 1 grading on evaluation of neurosurgery residency applicants in the United States: a program director survey	LORs, research, Step 2, class rank, and away rotations increased in importance. Most believe Step 1 predicts ABNS pass rates; few think change improves clinical preparedness
2021	Romano et al. [3]	Optimizing the residency application process: insights from neurological surgery during the pandemic virtual application cycle	Virtual interviews increased applications, sessions, and interviews. Most said virtual interviews worsened the assessment of “fit.” Preferred hybrid format; supported preference signaling and interview caps
2022	Jimenez et al. [4]	Perceptions of the virtual neurosurgery application cycle during the coronavirus disease 2019 (COVID-19) pandemic: a program director survey	More applications, sub-internship performance, LORs, and Step 1 top factors. Many want virtual interviews to remain for cost savings, but feel it disadvantages DOs and IMGs
2004	Lunsford et al. [12]	Survey of United States neurosurgical residency program directors	Most programs expressed concern over an 80-hour work week detracting from their training program. Interest in reassessment of the method of training neurosurgical residents, especially with regard to manpower needs. Most PDs thought that there were too few current residents at their institution and too few practicing neurosurgeons in the U.S. The majority of PDs have the perception of the beginning of or current decline in interest of U.S. medical students in neurosurgery. Most respondents indicated that current residency review committee governance and the committee's relationship with PDs were satisfactory.
2005	Cohen-Gadol et al. [13]	Resident duty hours reform: results of a national survey of the program directors and residents in neurosurgery training programs	By July 2003, most programs reported implementing ACGME work requirements and the employment of ancillary healthcare professionals to fulfill the reform. Most PDs feel that the ACGME guidelines have negatively impacted resident training experience and patient care continuity. The majority feel that residents are less familiar with patients, that trauma care has been diluted by fewer night calls, and that chief residents operate on fewer complex cases after ACGME restrictions. Few think that work hour limitations would increase research/publications for residents, that ABNS test scores would increase, or that attendance at national conferences would increase. Most programs employed ancillary healthcare professionals to decrease work hours and do not believe that this hiring has limited clinical experience for residents.
2013	Ganju et al. [15]	The role of simulation in neurosurgical education: a survey of 99 United States neurosurgery program directors	Most PDs believe simulation improves outcomes and skills, especially under work-hour restrictions, but not a replacement. Many support making simulation integral and mandatory in training
2014	Daniels et al. [14]	The current state of United States spine surgery training: a survey of residency and spine fellowship program directors	Residents graduate with >450 spine cases and high confidence in decompression/instrumentation. Most require a fellowship for deformity surgery. PDs support simulation and competency-based training
2014	Kshettry et al. [17]	The role of laboratory dissection training in neurosurgical residency: results of a national survey	Most programs use cadaveric dissection (cranial/spine focus) and view it as more beneficial than simulation. Strong support for a universal dissection curriculum
2015	Haglund et al. [18]	Difficult conversations: a national course for neurosurgery residents in physician-patient communication	Many PDs indicated that residents lack sufficient skill in delivering bad news and outcome discussions. Strong support for online communication modules in national boot camps
2018	Gupta et al. [19]	Neurosurgical resident error: a survey of U.S. neurosurgery residency training program directors’ perceptions	Procedural errors most common; often not lasting injury, but some require repeat surgery. Few programs have formal error reporting/remediation protocols
2019	Limoges et al. [20]	Pediatric neurosurgery training during residency in the United States: a program director survey	Resident pediatric neurosurgery training varies across US programs. Most programs have 4 or fewer pediatric neurosurgeons, dedicated pediatric rotation most commonly 4-6 months in length, usually at freestanding children’s hospitals
2019	Lepard et al. [21]	Neurosurgical resident research education: a survey of United States residency program directors	Nearly all programs hold regular journal clubs aimed at fostering critical appraisal skills. About half of the programs have form EBM curriculum. Annual publication requirements exist at just over half of programs, and higher resident publication output correlates with greater protected research time, trained journal club facilitators, and earlier research involvement
2020	Miller et al. [22]	International electives in neurological surgery training: a survey of program directors from Accreditation Council for Graduate Medical Education-approved neurological surgery programs	Fewer programs offer international electives. Many PDs express interest despite barriers such as funding, approval processes, call conflicts, and partner establishment. PDs value these rotations for enhancing cultural awareness, exposure to unique pathologies, and diagnostic/clinical skills. Perceived value was highest among those with existing electives
2023	Kilbourn et al. [16]	Incorporating simulation into the neurosurgical residency curriculum: a program director survey	Only about half of the programs formally integrate simulation. Common barriers are cost and space. Programs utilizing simulation find it effective for technical and anatomic skills, but less so for non-technical skills. Human cadavers are most commonly used. Most programs provide feedback via verbal debriefing, and COVID-19 did not substantially change simulation use
